# Context-dependent transcriptional regulations of YAP/TAZ in stem cell and differentiation

**DOI:** 10.1186/s13287-021-02686-y

**Published:** 2022-01-10

**Authors:** Juan Luo, Peng Li

**Affiliations:** 1 Scientific Research Center, The Seventh Affiliated Hospital of Sun Yat-Sen University, Shenzhen, 518107 Guangdong People’s Republic of China; 2 Guangdong Provincial Key Laboratory of Digestive Cancer Research, The Seventh Affiliated Hospital of Sun Yat-Sen University, No. 628 Zhenyuan Road, Shenzhen, 518107 Guangdong People’s Republic of China

**Keywords:** Hippo pathway, YAP/TAZ, Context-dependent, Transcriptional output, Stem cell and differentiation

## Abstract

Hippo pathway is initially identified as a master regulator for cell proliferation and organ size control, and the subsequent researches show this pathway is also involved in development, tissue regeneration and homeostasis, inflammation, immunity and cancer. YAP/TAZ, the downstream effectors of Hippo pathway, usually act as coactivators and are dependent on other transcription factors to mediate their transcriptional outputs. In this review, we will first provide an overview on the core components and regulations of Hippo pathway in mammals, and then systematically summarize the identified transcriptional factors or partners that are responsible for the transcriptional output of YAP/TAZ in stem cell and differentiation. More than that, we will discuss the potential applications and future directions based on these findings.

## The core components of Hippo pathway

**Fig. 1:**
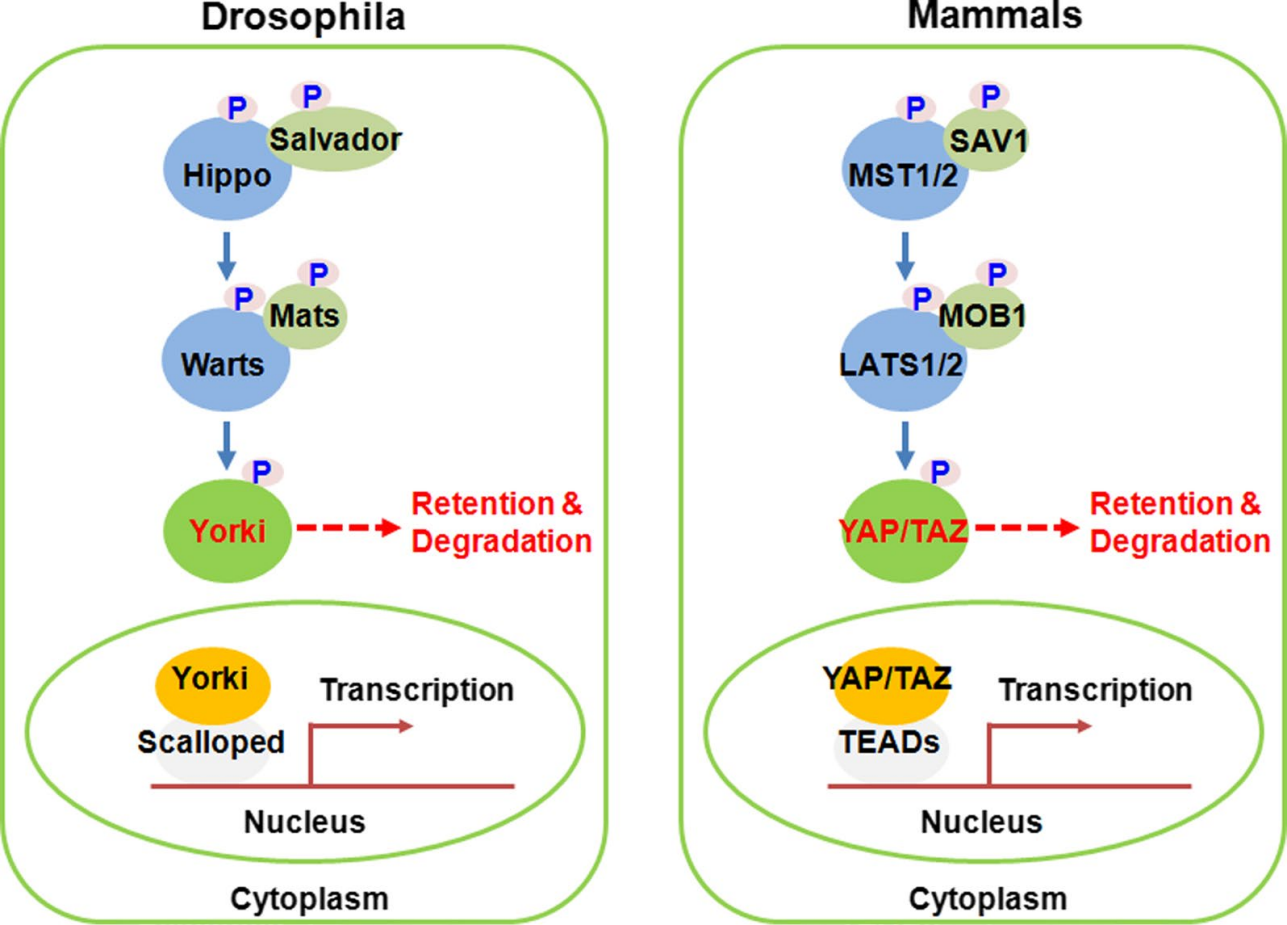
Hippo pathway components in Mammals and Drosophila

The initial identification of Hippo pathway was in *Drosophila melanogaster* by genetic mosaic screens for tumor suppressor genes, and the subsequent researches by molecular and genetic studies have validated some highly conserved aspects of this pathway in mammals, including the core components, regulation mechanisms and its functional role in organ size control. Genetic inactivation of the genes in flies, including the NDR family protein kinase Warts (LATS in mammals) [1, 2], the WW domain-containing protein Salvador (SAV1 in mammals) [3, 4], the Ste20-like protein kinase Hippo (MST in mammals) [5–9] and the adaptor protein Mob-astumor-suppressor (MOB in mammals) [10], resulted in an overall similar phenotype with tissue overgrowth characterized by increased cell proliferation and reduced cell death. The subsequent biochemical studies revealed these tumor suppressors could form a kinase cascade in which the Hippo-Salvador kinase complex (MST1/2-SAV1 in mammals) directly phosphorylated and activated the Warts-Mob kinase complex (LATS1/2-MOB1 in mammals) [9, 11]. Later, the transcriptional coactivator Yorkie (YAP/TAZ in mammals) was identified to be the downstream effector of this kinase cascade in growth regulation [12], via binding with transcription factor Scalloped (TEAD in mammals) [13–16]. A summary of these core components in drosophila and mammals is shown in Fig. 1.

## The regulations of Hippo pathway

In mammals, MST1/2 could directly phosphorylate MOB1 and LATS1/2 at the hydrophobic motif (Lats1 T1079 and Lats2 T1041), which were required for the phosphorylation of LATS activation loop (Lats1 S909 and Lats2 S872), and thereby an increased LATS kinase activity [17]. LATS activation further enhanced its interaction with YAP/TAZ via its PPxY motifs and the WW domains of YAP/TAZ, by which YAP was directly phosphorylated at five serine residues, including Ser61, 109, 127, 164 and 397 [18–20]. Among these residues, YAP Ser127 and Ser397 phosphorylations were mainly responsible for the suppressive role of Hippo kinase in cell proliferation and organ growth. Specifically, phosphorylation of YAP at Ser127 enhanced its binding with 14-3-3 proteins, thereby the subsequent sequestration of YAP in the cytoplasm. While the YAP phosphorylation at Ser397 would facilitate its sequential recognition by CK1 kinase and the E3 ligase SCF^β-TRCP^ for YAP ubiquitination and degradation [21]. Therefore, Hippo kinase cascade-mediated YAP/TAZ regulation represents a central regulatory mechanism for cell proliferation and organ growth in mammals.

## The regulators of Hippo pathway

**Fig. 2:**
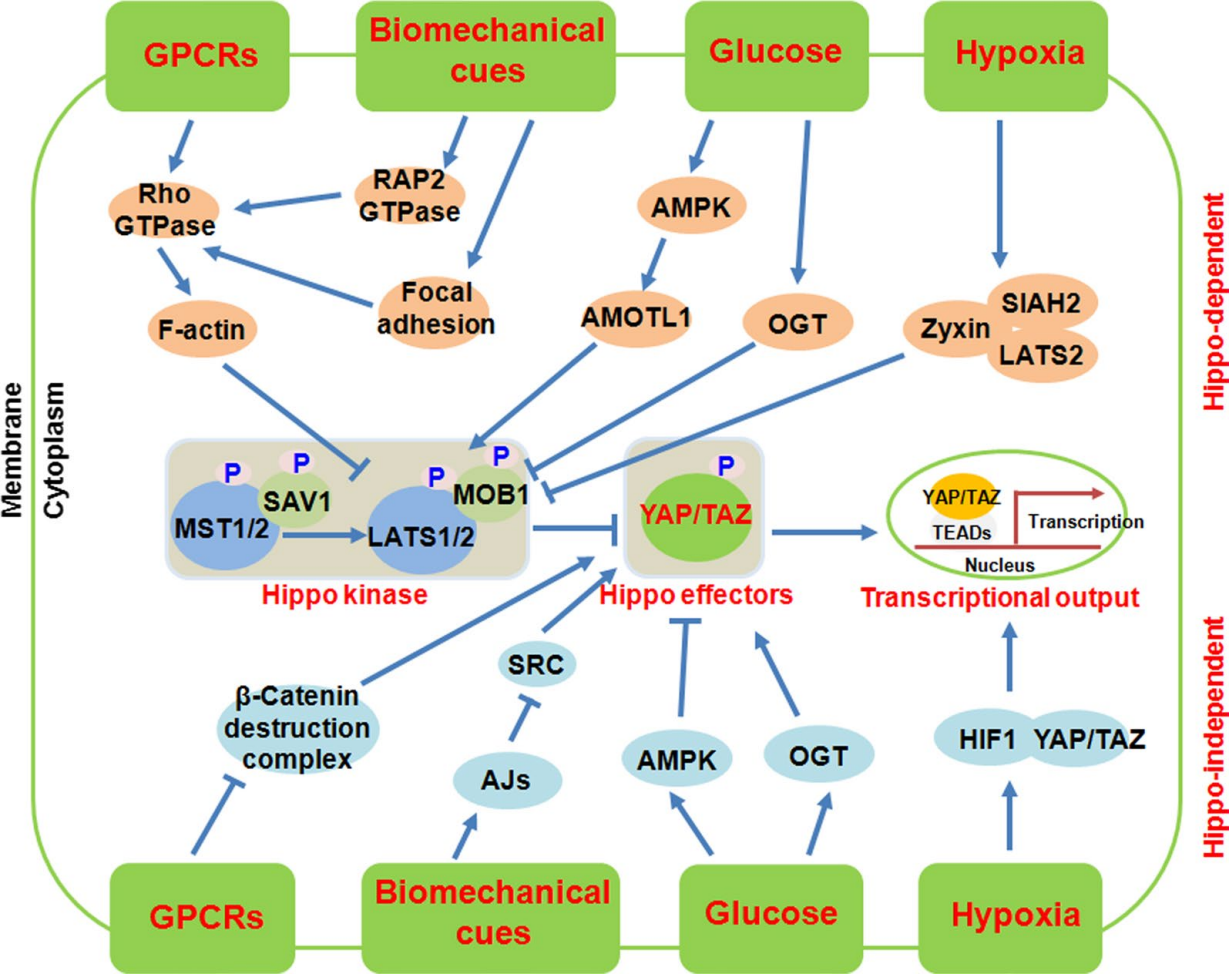
The regulations and upstream regulators of Hippo pathway in Mammals

Since the discovery of Hippo-YAP/TAZ signalling pathway in mammals, the researchers were trying to identify the upstream regulators to clarify how this pathway was initiated, and then regulated various physiological functions. At least so far, this pathway was participated in the regulations of development, tissue regeneration and homeostasis, inflammation, immunity and cancer. To this end, in the past two decades, a large number of upstream regulators of Hippo-YAP/TAZ pathway have been identified, including growth factors and hormone, glucose, hypoxia and biomechanical cues. Dysregulation of these regulators has been implicated in various diseases and cancers. In this part, we will give a brief overview on how these upstream regulators integrate the extracellular signals together with YAP/TAZ activity in mammals (Fig. 2).

### Growth factors and hormone

G protein-coupled receptors (GPCRs) are a large family of cell-surface receptors characterized by seven transmembrane helical domains. Upon binding to their cognate ligands, such as the growth factors and hormone, intracellular signals are activated and transduced through their heterotrimeric G-proteins. Yu et al. have discovered that GPCRs-induced G_12/13_, G_q/11_, and G_i/o_ activation could inhibit LATS1/2 phosphorylation, thus leading to increased nuclear translocation of YAP/TAZ. Conversely, GPCRs that activate G_s_ signalling enhanced the phosphorylation of LATS1/2, leading to increased YAP/TAZ sequestration within the cytosol. Mechanistically, the regulation of Hippo-YAP/TAZ by GPCRs is mediated by Rho GTPases and the remodeling of actin cytoskeleton [22]. Moreover, consistent with these findings, YAP/TAZ were identified to be important oncogenic drivers and therapeutic targets of uveal melanoma, in which hyperactive mutations of Gα_q/11_ are dominant in patients [23–25].

### Glucose

Extracellular nutrition signal is an important regulator of Hippo-YAP pathway. Zhang et al. have found that high glucose stimulation could induce YAP OGlcNAcylation at Thr241 by O-GlcNAc transferase (OGT) in a LATS-independent manner, whereby YAP protein stability and transcriptional activity were enhanced [26]. In addition, OGT-mediated YAP OGlcNAcylation could also happen at serine 109, by which LATS-YAP interaction was disrupted and YAP transcriptional activity was activated [27]. These studies highlighted that YAP OGlcNAcylation played a critical role in high-glucose-stimulated cell growth and tumorigenesis. In addition, Hippo pathway-independent mechanisms are also involved in this process. In the presence of glycolysis, phosphofructokinase-1 (PFK1), a key glycolytic enzyme, could bind with TEAD1 to stabilize a PFK1-TEAD1-YAP protein complex in the nucleus, revealing a molecular mechanism for PFK1-induced tumor malignant in breast cancer [28]. Conversely, removal of glucose or inhibition of glycolysis with 2-deoxyglucose have activated both the Hippo kinase and AMP-activated protein kinase (AMPK). Both of them could inhibit YAP activity either through the AMOTL1-mediated LATS activation, or AMPK-induced YAP phosphorylation at Ser94, thereby inhibiting the cell proliferation and tumor growth [29–31]. Taken together, these studies demonstrated that Hippo-YAP signalling played an essential role for coordinating energy status with cell proliferation.

### Hypoxia

Hypoxia represents a common feature of the solid tumors, and the genes associated with angiogenesis and cell survival will be activated in response to oxygen deprivation to maintain tumor cell proliferation. Ma et al. have reported that SIAH2, an ubiquitin E3 ligase, directed LATS2 to degradation via the ubiquitin–proteasome pathway under low oxygen, whereby YAP nuclear translocation and transcriptional activity were enhanced [32]. Further studies revealed the secretion of transforming growth factor beta (TGFβ) was increased under hypoxia, which in turn stabilized the ternary complexes, consisting of Zyxin, SIAH2 and LATS2, thereby facilitating SIAH2-mediated LATS2 degradation and reducing LATS2-dependent YAP phosphorylation [33]. In addition, hypoxia-inducible factors (HIFs)-induced gene expression, including *VEGFA*, *IGF1* and *LOX*, was thought to be the hallmark of hypoxia [34]. TAZ has been found to function as a coactivator of HIF1, via direct interaction with the transactivation domain of HIF1A, by which TAZ could direct HIF1 to the hypoxia response elements and contribute to the transcription of downstream genes, thereby promoting tumor cell survival and migration [35]. Collectively, these studies indicated that YAP/TAZ was a key nexus between hypoxia and malignant tumor phenotypes, and which may offer promising targets for intervention in malignant cancers displaying intratumoral hypoxia.

### Biomechanical cues

Biomechanical cues refer to the mechanical forces generated by cell interaction with its microenvironment, including the extracellular matrix, shear forces and adjacent cells. The initial finding that YAP phosphorylation and subcellular localization were regulated by cell–cell contact and cell density [18], has stimulated the researchers to realize that the extracellular mechanical forces may play key roles in orchestrating organ/tissue growth and homeostasis. Indeed, the subsequent studies showed that spreading morphology of cells at low density, or exposure cells to stiff matrices activated YAP, whereas compact morphology at high cell density, or shift cells from stiff to soft matrices inhibited YAP activity. Further studies revealed that actin cytoskeleton, as well as its downstream regulators Rho family GTPases and small GTPase RAP2, were important mediators to transduce mechanical cues to Hippo-YAP signalling [36–38]. a-Catenin is a component of adherens junctions to link the membrane cadherins and actin cytoskeleton, disruption of a-catenin in skin keratinocytes lost the cell–cell contact inhibition and caused squamous cell carcinoma. Further studies showed a-catenin inhibited Yap activity and tumour progression through constitutively anchoring Yap to adherens junctions, or directly inhibiting an Itgb4-Src-Yap signalling pathway [39–41]. More interestingly, Wang et al. found that dynamic mechanical forces generated by blood flow regulated YAP/TAZ activity in endothelial cells, connecting YAP/TAZ regulation by mechanotransduction to atherosclerosis. Specifically, atheroprotective unidirectional shear stress inhibited endothelial YAP/TAZ due to inhibition of the Integrin-Gα_13_-RhoA activity, while the atheroprone disturbed flow activated YAP/TAZ, which in turn enhanced JNK signaling and induced the expression of proinflammatory genes. Moreover, endothelial YAP/TAZ knockdown or MnCl2 treatment delayed atherogenesis, indicating that YAP/TAZ could become a potential therapeutic target against atherosclerosis [42].

## Transcriptional regulations of YAP/TAZ in stem cell and differentiation

Due to the lack of any DNA-binding domain, YAP/TAZ act as a coactivator and rely on their binding to TEAD family members to mediate their transcriptional output. Even so, YAP-TEAD complex is not sufficient to execute all transcriptional programs in different contexts. To this end, a large amount of YAP/TAZ-interacting partners are being gradually identified, including transcription factors and epigenetic modifiers, and all of them have proved to play pivotal roles for YAP/TAZ-associated transcriptional outputs and biological functions. In this part, we aim to systematically summarize the identified YAP/TAZ-interacting partners so far that are responsible for the downstream transcriptional outputs of YAP/TAZ in stem cell and differentiation. Here, we mainly focus on the roles of YAP/TAZ in the blastocyst/embryonic stem cells and adult stem cells (Table 1).

**Table 1:** Context-dependent transcriptional regulations of YAP/TAZ in stem cell and differentiation

System	Interacting partners	Context	Regulated target genes	Tissue/cell types	Functions	Main reference
Blastocyst	TEAD	Embryo development	*Cdx2*	ESCs and embryos	To distinguish the TE and ICM	[45]
			*Oct4* and *Nanog*	ICM	To induce the epiblast lineage cells	[47]
ESCs	TEAD and P300	ESC differentiation	*Mcl1, Bcl2* and *Bcl2l1*	Mouse ESCs	To promote cell survival during ESC differentiation	[48]
	TEAD and P73	RASSF1A-induced stem cell differentiation	*OCT4* and differentiation-associated genes	Mouse ESCs	Function as a “switch” between pluripotency and initiation of differentiation	[49]
	TEAD, SMAD2/3, OCT4, NuRD and FOXH1	TGF-β signalling-mediated ESC pluripotency maintenance and mesoderm specification	*NANOG and*: *EOMES*	Human ESCs	To direct discrete SMAD2/3 signalling outcomes in the context of pluripotency and mesoderm induction	[50]
NSCs	TEAD and SMAD1/4	BMP2 activation in mouse embryonic NSCs	*Ccnd1*	Mouse embryonic NSCs	To represses the proliferation of embryonic NSCs	[53]
	SMAD1/5	Radial glia cell self-amplification in the developing cerebral cortex	–	Radial glia cells	To promote the radial glia cell self-amplification in embryos, and prevent their premature	[54]
	SMAD1/4/8	BMP2-induced neocortical astrocytic differentiation	Astrocytic differentiation-associated genes	NSCs and astrocytes	To induce the astrocytic differentiation in the developing mouse neocortex	[55]
	Pax3	Neural crest development	*Mitf* and *Myf5*	Premigratory neural crest cells	Essential for neural crest delamination during embryonic development	[56]
	TEAD and SOX10	Schwann cell proliferation and myelination	*Gnas* and myelination genes	Peripheral nerves system and Schwann cells	To promote Schwann cell proliferation and myelination	[57]
MSCs and SSCs	RUNX2	Src activation-mediated osteogenic differentiation	Bone-specific osteocalcin gene	Osteoblasts	To repress osteoblast differentiation	[60]
	AP2a and RUNX2	Osteogenic differentiation and bone regeneration	*BARX1*	MSCs	Inhibition of osteoblast differentiation	[61]
	TEAD and RUNX2	Osteoblast differentiation and bone development	*Alp*, *Cola1*, and *Osterix*	MSCs and osteoblasts progenitor	Inhibition of osteoblast differentiation	[62]
	TEAD and Snail/Slug	SSCs differentiation	*Ctgf*, *Ankrd1*, *Axl*, *Dkk1* and *Cyr61*; *Bglap2*, *Osterix* and *Alp*	SSCs	To regulate SSC proliferation and osteogenic differentiation	[64, 65]
Cardiac cells	β-Catenin	Hippo-deficient embryo hearts	*Sox2* and *Snail2*	Embryonic cardiomyocyte	To regulate cardiomyocyte proliferation and heart size	[68]
	Myb-MuvB (MMB) complex	Loss of the Hippo-signalling component SAV1	*TOP2A*, *CDC20*, *CENPF* and *AURKA*	Embryonic cardiomyocyte	To promote cardiomyocyte mitosis and proliferation	[69, 70]
	FoxO1	Oxidative stress response	*Catalase* and *MnSOD*	Cardiomyocytes	To promote cell survival in response to oxidative stress	[72]
	TFEB	Lysosomal storage diseases	*MAPLC3B*	LSD mouse model	To promote autophagic and lysosomal gene expression	[74]
ISCs	TEAD and Klf4	In the intestinal epithelium expansion and differentiation	*Muc2*, *Rps26*	Mouse intestine	To regulate ISC proliferation and differentiation to goblet cells	[76]
	TLE	Dual inhibition of TEAD-mediated transcriptional and LATS activities in ISCs	*Axin2* and *Lgr5*	ISCs	To block Wnt/TCF-mediated transcription in ISCs	[78]
Pancreas	TEAD and Pancreatic-TFs	Pancreas development	*SOX9*, *HHEX* and *MNX1*	Human embryonic pancreas and ESC-derived progenitors	To promote the outgrowth of pancreatic multipotent progenitor cells	[79]
Liver	TEAD and PPARα	Liver growth and regeneration	*CTGF*, *CYR61*, *ANKRD1*; *AXOX1* and *CYP4A*	Mouse live and hepatocyte	To promote liver growth and regeneration	[81]

### Role of YAP/TAZ in the blastocyst/embryonic stem cells

#### Blastocysts

The first cell fate specification from the blastocyst is trophoblast lineage, which surrounds the inner cell mass (ICM) to form the trophectoderm (TE) [43]. The ICM further differentiates into the epiblast and primitive endoderm at the late blastocyst stage [44]. Coordination of these cell specifications is essential for early embryo development. Nishioka et al. found that Yap-Tead4 module localized in the nuclei of outside cells at the blastocyst stage will induce *Cdx2* expression, a TE-specific transcription factor, and repress the expression of ICM-specific pluripotency genes, such as *Oct4* and *Nanog*, thereby promoting TE specification. While in inner cells, Yap is phosphorylated and sequestered in cytoplasm due to the cell-contact inhibition, whereby its transcriptional activity is repressed [45]. Inactivation of upstream core components of Hippo pathway in ICM, such as Lats1, will lead to Yap/Cdx2 activation and ICM transformation into the TE lineage [46]. These studies revealed that cell position influenced the cell fate specification in preimplantation embryos by regulating subcellular localization of Yap. However, during the epiblast formation process from ICM, high YAP-TEAD activity is required for the strong expressions of pluripotency factors and the induction of an epiblast with naive pluripotency [47], uncovering a time window-dependent YAP-TEAD activity during this stage of embryonic development.

#### Embryonic stem cells (ESCs)

ESCs, isolated from the ICM in vitro, can propagate in vitro and differentiate into all adult cells, they therefore provide useful materials for stem cell research with strong potential in regenerative medicine. LeBlanc et al. have showed that Yap1/Tead4 could complex with p300 to activate anti-apoptotic genes, and repress pro-apoptotic genes during in vitro ESC differentiation, thereby safeguarding ESCs from excessive apoptosis [48]. This study uncovered a differentiation-specific role for Yap1 in ESCs via recruitment of p300. Furthermore, Papaspyropoulos et al. identified the tumor suppressor RASSF1A as a key player to drive the early cell fate specification, via regulation of YAP-interacting partners in different contexts. Specifically, at ESC stage, RASSF1A expression is repressed, which will facilitate YAP/TEAD complexing with β-catenin/TCF3 module to occupy the Oct4 distal enhancer, thereby inducing pluripotency gene expression and maintaining stemness. Once the ESCs start for differentiation, RASSF1A will be upregulated, and which will promote a YAP/p73 transcriptional program to modulate the cell differentiation [49]. Therefore, this study revealed that RASSF1A functioned as a “switch” between pluripotency and initiation of differentiation by modulating YAP-interacting partners. In addition, in human ESCs, TGFβ-SMAD signaling is important for both pluripotency maintenance and mesoderm specification. Beyer et al. discovered that TAZ/YAP/TEAD, SMAD2/3 and OCT4 (TSO) collaborated with NuRD repressor complexes to buffer pluripotency gene while suppressing mesoderm gene expression in human ESCs. Upon the ESCs start to differentiating, TSO module will be disrupted and replaced with a SMAD-FOXH1 module to induce the mesoderm lineage cell specification [50]. This study demonstrated that YAP/TAZ-mediated switch elements directly controlled the TGF-β signaling outcomes in the contexts of pluripotency and mesoderm induction. Taken together, all these evidences proved that YAP/TAZ-interacting partners determined their functional transcription outputs in different contexts, which is pivotal for ESC maintenance and differentiation.

### Role of YAP/TAZ in the adult stem cells

#### Neural stem cells (NSCs)

NSCs, residing along the ventricle of the developing vertebrate neural tube, are responsible for giving rise to the vast numbers and diverse types of neurons and glia, which constitute the mature nervous system [51]. Therefore, coordination of NSC proliferation and differentiation is vital for normal nervous system development. BMP-SMAD pathway has emerged as critical regulators of NSC self-renewal and differentiation [52]. Yao et al. found that BMP2 treatment could inhibit mouse NSC proliferation, through reduction of YAP nuclear translocation, YAP/TEAD interaction, and YAP/TEAD-induced Cyclin D1 expression. Mechanistically, Smad1/4, the effectors of BMP2 signaling, competed with YAP for the interaction with TAED1, and thus inhibited YAP’s co-transcriptional activity [53]. This study revealed a potential role of YAP regulation in NSC proliferation. Similarly, Najas et al. showed that BMP-induced Smad1/5 activation could stimulate radial glial cell growth of the developing cerebral cortex, via a direct interaction with YAP and independently of TEAD family transcriptional factors [54]. However, during the astrocytic differentiation, YAP could stabilize SMAD1 and promote BMP2-induced neocortical astrocytic proliferation and differentiation, revealing a context-dependent role of YAP in NSC amplification and differentiation [55]. In addition, in premigratory neural crest, the Pax family member Pax3 could recruit Yap/Taz at the promoter regions to synergistically activate melanocyte gene expression, such as *Mitf*, and which was independent of Tead family factors, demonstrating a Pax3-depedent role of Yap/Taz in neural crest cell specification [56]. In peripheral nervous system, Schwann cell proliferation and myelination are essential for motor functions, and YAP/TAZ have been found to participate in these processes in a stage-dependent manner. Specifically, YAP/TAZ-TEAD module could induce Schwann cell proliferation by activating cell cycle genes and repressing *Gnas*. However, when the cells start for myelination, this module will be recruited by SOX10 to the enhancer regions and promoted the myelination-associated genes, including *Mbp*, *Mpz*, *Pmp22* and *Mal* [57]. Therefore, this study uncovered the dual roles of YAP/TAZ in Schwann cell proliferation and myelination via specifically interacting with TEAD and SOX10 respectively. Taken together, all these studies reflected a context-dependent role of YAP/TAZ in neural lineage cells by complexing with different partners.

#### Mesenchymal stem cells (MSCs) and skeletal stem cells (SSCs)

MSCs are a specialized population of progenitor cells that are positioned throughout host tissues during the lifespan [58]. In particular, bone marrow-derived MSCs (also referred to as SSCs) are able to differentiate into osteoblasts, chondrocytes and adipocytes [59]. They therefore hold great promise for studying osteoblastogenesis and guiding bone regeneration. The Runx family member 2 (Runx2) is a target of several extracellular signals that regulate osteoblast formation and homeostasis in vivo. Zaidi et al. found that Src/Yes-mediated YAP phosphorylation facilitated its recruitment by Runx2 to subnuclear sites and repressed the bone-specific osteocalcin genes. Interference with the Src-YAP-Runx2 pathway at any level could rescue the osteocalcin gene expression, thereby revealing a repressive role of YAP in osteoblast formation [60]. Recently, Lin et al. showed that transcription factor AP2a could compete with RUNX2 to bind YAP, thereby releasing the inhibition of YAP to RUNX2 by forming a YAP-AP2a protein complex in MSCs. Further studies showed that YAP/AP2a complex subsequently moved to *BARX1* promoter region and inhibited its transcription, thereby enhancing the MSC-induced osteogenic differentiation [61]. This study uncovered new mechanisms for MSC-induced osteogenic differentiation and shed light on the bone regeneration. Likewise, Suo et al. found that vestigial-like family member VGLL4 played a similar role with RUNX2 in osteoblast differentiation through a direct interaction with TEADs, by which RUNX2-TEADs interaction was inhibited, and then the inhibitory effect of TEADs on RUNX2 was relieved [62]. Owing to the tissue affinity, SSCs are the most classic used seed cells for bone regeneration [63]. Therefore, dissecting its mechanisms of self-renewal and differentiation will help in improving the results of bone tissue engineering. Tang et al. found that zinc-finger transcription factors, Snail and Slug, regulated SSC proliferation and differentiation by forming complexes with YAP/TAZ or RUNX2 in different contexts, thereby playing dual roles in SSC self-renewal and osteogenic differentiation. In particular, Snail/Slug complexing with YAP/TAZ could promote SSC proliferation, but instead that Snail/Slug recruitment by RUNX2 will lead to the osteogenic differentiation [64, 65]. Collectively, all these findings identified that YAP-TEAD module play a repressive role in regulating osteoblast differentiation and bone development. Therefore, releasing its repressive effect on RUNX2 may be a strategy for improving osteogenic differentiation and enhancing bone regeneration.

#### Cardiomyocyte

Cardiomyocyte proliferation is essential for heart growth and regeneration, multiple studies have identified YAP as an important regulator of cardiomyocyte proliferation [66, 67]. However, the detailed mechanisms have long remained unclear. Heallen et al. firstly reported that Yap interacted with β-catenin on *Sox2* and *Snai2* gene promoters to control the cardiomyocyte growth, uncovering a nuclear interaction between Hippo and Wnt signaling effectors that could restrain cardiomyocyte proliferation and control heart growth [68]. Furthermore, Grundl et al. demonstrated that YAP activation-induced cardiomyocyte proliferation was dependent on the Myb-MuvB (MMB) complex, by which YAP regulated a set of cell cycle genes in cardiomyocytes [69, 70], highlighting MMB as a critical downstream effector to mediate YAP-induced cardiomyocyte proliferation and heart regeneration. In addition, activation of YAP also promoted myocardial regeneration after myocardial infarction. Ischemia/reperfusion has been reported to cause myocardial injury and cardiac dysfunction, through production of reactive oxygen species (ROS) and cardiomyocyte death [71]. In this process, FoxO family transcription factor FoxO1, has been found to recruit YAP and regulate antioxidant gene expression, like *Catalase* and *MnSOD*, in a Hippo-dependent manner, thereby reducing oxidative stress and promoting cardiomyocyte survival [72]. Lysosomal storage disorder (LSD) was characterized by accumulation of damaged proteins and organelles in cells and functional abnormalities in major organs [73]. YAP has been observed to accumulate in RagA/B conditional knockout mousehearts, an LSD model in which lysosomal acidification is impaired irreversibly. Further studies revealed that YAP physically interacted with transcription factor EB (TFEB), a master transcription factor that controls autophagic and lysosomal gene expression, thereby facilitating the accumulation of autophagosomes without degradation. Inhibition of YAP ameliorated cardiac hypertrophy and contractile dysfunction, revealing a critical role of YAP in the development of cardiomyopathy in LSD [74]. These findings overall revealed that activation of YAP-mediated transcriptional activity may be useful for promoting cardiac regeneration under both normal physiological and pathological conditions.

#### Intestine stem cells (ISCs)

The ISCs located in the crypt base are responsible for the generation of rapidly self-renewing epithelium lining the surface of intestine, including enterocytes, goblet, enteroendocrine and paneth cells [75]. Imajo et al. found that YAP/TAZ promoted both the proliferation of ISCs and their differentiation into goblet cells, via binding with two different types of transcription factor. Specifically, YAP/TAZ complexed with TEAD transcription factors could promote ISC proliferation, and on receiving differentiation cues, YAP/TAZ cooperated with Klf4 to promote their differentiation into goblet cells, uncovering the dual roles of YAP/TAZ in ISC self-renewal and differentiation in different contexts [76]. Besides, Wnt pathway is a master regulator for ISC self-renewal and differentiation [77]. Li et al. found that Lats1/2 are essential to maintain Wnt pathway activity and ISC identity, and deletion of Lats1/2 kinases abolished ISCs but induced the Wnt-uncoupled crypt expansion. Further studies revealed that an interaction between YAP/TAZ and Groucho/TLE repressors was responsible for the inhibition of Wnt/TCF-mediated transcription in intestinal epithelium [78].

#### Pancreatic and hepatic progenitors

Understanding the pancreas development has pivotal implications for pancreatic regeneration and diabetes. Using human embryonic pancreas and hESC-derived pancreatic progenitor cells, Cebola et al. identified some stage-specific transcripts and associated enhancers were co-occupied by transcription factors that are essential for pancreas development. Further investigations showed YAP/TEAD module functioned as a regulatory switch to activate stage-specific transcriptional program in pancreatic progenitor cells [79]. This work therefore uncovered a central role of YAP/TEAD as signal-responsive regulators of multipotent pancreatic progenitors, and provided a resource for the study of embryonic development of the human pancreas.

The liver has a tremendous capacity to regenerate after injury induced by toxin, surgical resection or infection, and this process is a tightly controlled and regulated by complex signaling pathways. Peroxisome proliferator-activated receptor α (PPARα), a ligand-activated nuclear receptor, plays an important role in liver regeneration, but the underlying mechanisms remain largely unclear [80]. Recently, Fan et al. found that PPARα activation promoted hepatocyte hypertrophy and proliferation through regulating the expression of YAP and its downstream targets. Further studies showed that PPARα could directly bind to YAP and induce its nuclear translocation, thereby promoting YAP/TEAD-mediated transcriptional output and hepatocyte proliferation [81]. This study implicated a positive regulatory role of PPARα in liver development and regeneration, which may serve as potential target for manipulating liver size and regeneration.

## Conclusion and future perspectives

The Hippo pathway, initially identified as a critical regulator of cell proliferation and organ size, has received a surge of interest in the last two decades. Accumulating evidences thus far highlighted the roles of this pathway in organ development, tissue regeneration and stem cell determination. In particular, extensive genetic studies using the mouse models have revealed that Hippo-YAP/TAZ signaling played a pivotal role and had a broad function in mammalian development in most of the tissues and organs. As the downstream effectors, YAP/TAZ often need to collaborate with other DNA binding factors to regulate the developmental programs in different biological contexts. Therefore, identifying these key DNA binding factors and uncovering the regulation mechanisms in different contexts, such as in pluripotent and adult stem cells, will be beneficial for the stem cell research and regeneration medicine in future. Moreover, dysregulation of the Hippo pathway has also been observed in various cancers [82, 83], and therefore, targeting of this pathway represents a very promising strategy for cancer treatment. However, the issue as we discussed in this review is also existed in YAP/TAZ-associated cancers. Owing to lack of a DNA-binding domain, YAP/TAZ thus are dependent on other transcription factors to mediate their transcriptional output. Indeed, growing evidences have suggested that the functions of YAP/TAZ in cancer development are finely tuned in different contexts via interaction with different partners. For example, despite that YAP/TAZ complexing with TEADs orchestrated many processes during oncogenesis through their co-transcriptional activation activity, many more other YAP/TAZ-interacting partners are being identified gradually to execute distinct functions. Hoxha et al. found that YAP/TAZ could also act as transcription repressors through interacting with transcription factor YY1 and polycomb repressive complex member EZH2, by which a broad network of genes mediating the cell hyperproliferation were transcriptionally repressed [84]. This study revealed a transcriptional repressive role of YAP/TAZ in tumorigenesis. Moreover, YAP/TAZ were also found to play a tumor-suppressive role via interacting with p73, a homolog of p53, thereby promoting p73-dependent apoptosis and *BAX* gene expression in response to DNA damage [85–88]. Taken together, these studies overall provided evidences that YAP/TAZ played a versatile role in tumorigenesis via complexing with distinct partners. Therefore, identifying the key partners that are responsible for YAP/TAZ-mediated transcriptional regulations in different contexts of cancer development is still imperative to realize the precise diagnosis and treatment of cancer.

## Data Availability

The datasets used and analyzed in this study are available from the corresponding author on reasonable request.

## Abbreviations

AMPK: AMP-activated protein kinase
AP2a: Transcription factor AP-2 alpha
ESCs: Embryonic stem cells
GPCRs: G protein-coupled receptors
Hifs: Hypoxia-inducible factors
ICM: Inner cell mass
LATS1/2: Large tumor suppressor 1/2
LSD: Lysosomal storage disorder
MAML: Nuclear effector Mastermind-like
MOB1: MOB kinase activator 1
MMB: Myb-MuvB complex
MST1/2: STE20 like protein kinase 1/2
MSCs: Mesenchymal stem cells
NSCs: Neural stem cells
OGT: O-GlcNAc transferase
PFK1: Phosphofructokinase-1
PPARα: Peroxisome proliferator-activated receptor α
ROS: Reactive oxygen species
Runx2: Runt related transcription factor 2
SAV1: Salvador homologue 1
SMAD1/4/5: SMAD family member 1/4/5
SSCs: Skeletal stem cells
TAZ: WW domain-containing transcription regulator protein 1
TEAD: TEA domain family member
TE: Trophectoderm
TFEB: Transcription factor EB
TGFβ: Transforming growth factor beta
TSO: TAZ/YAP/TEAD, SMAD2/3 and OCT4 complexes
VGLL4: Vestigial-like family member 4
YAP: YES-associated protein
